# Evaluation of a Newly Identified Endophytic Fungus, *Trichoderma phayaoense* for Plant Growth Promotion and Biological Control of Gummy Stem Blight and Wilt of Muskmelon

**DOI:** 10.3389/fmicb.2021.634772

**Published:** 2021-03-05

**Authors:** Wipornpan Nuangmek, Worawoot Aiduang, Jaturong Kumla, Saisamorn Lumyong, Nakarin Suwannarach

**Affiliations:** ^1^Faculty of Agriculture and Natural Resources, University of Phayao, Muang Phayao, Thailand; ^2^Department of Biology, Faculty of Science, Chiang Mai University, Chiang Mai, Thailand; ^3^Research Center of Microbial Diversity and Sustainable Utilization, Chiang Mai University, Chiang Mai, Thailand; ^4^Academy of Science, The Royal Society of Thailand, Bangkok, Thailand

**Keywords:** biocontrol, cucurbit, fungal endophyte, fungal disease, plant growth promotion

## Abstract

Gummy stem blight and wilt are known to cause enormous losses to the global production of muskmelon (*Cucumis melo*). In this study, the potential of endophytic fungi isolated from leaves of Siam weed (*Chromolaena odorata*) was investigated for the inhibition of mycelial growth of *Stagonosporopsis cucurbitacearum* and *Fusarium equiseti*. Twenty-one fungal isolates were obtained. The results indicated that a fungal isolate UP-L1I3 displayed the highest percentage in terms of inhibition of the mycelial growth of *F. equiseti* and *S. cucurbitacearum* at 90.80 and 81.60%, respectively. Consequently, this isolate was selected for its potential ability to promote plant growth and control gummy stem blight and wilt in muskmelon seedlings. Morphological and multilocus phylogenetic analyses revealed that the isolate UP-L1I3 was a new species that has been described herein as *Trichoderma phayaoense*. Pathogenicity test confirmed that *F. equiseti* and *S. cucurbitacearum* were the cause of gummy stem blight and wilt disease in muskmelon seedlings, respectively. However, no disease symptoms were observed in seedlings inoculated with *T. phayaoense*. It was found that *T. phayaoense* could be used preventively in muskmelon seedlings that were inoculated with *F. equiseti* and *S. cucurbitacearum*, which could then reduce the impact on the disease severity index. *T. phayaoense* was also effective in improving plant development by increasing plant height, as well as shoot and root dry weight values. Moreover, *T. phayaoense* could effectively increase weight, diameter, and the circumference and total soluble solid of fruit without having a negative effect on fruit quality parameters. Additionally, *T. phayaoense* was able to tolerate a commonly applied fungicide (metalaxyl) in recommended dosages for field applications.

## Introduction

Muskmelon (*Cucumis melo* L.) is one of the most popular edible fruits in the world. This fruit is rich in nutrients, minerals, and several other health-bioactive compounds that are beneficial to humans (Ismail et al., 2010; Parle and Singh, 2011). In 2018, the largest producer of melons (including muskmelons and cantaloupes) is produced in China at 1.27 million tonnes with global production at 2.73 million tonnes (FAOSTAT, 2020). Indonesia is the Southeast Asia’s leading melon producer, followed by the Lao People’s Democratic Republic and the Philippines. Nowadays, muskmelon production is an important economic activity in Thailand and the production area continues to increase. On the other hand, the incidence and severity of certain diseases have also increased when plantings occur at unsuitable locations. Plant pathogenic microorganisms (bacteria, fungi, and viruses) are known to cause diseases in muskmelons under the field before they are harvested, during harvesting and during storage, all of which can cause considerable yield loses (Walcott et al., 2004; Malik et al., 2010; Kehinde, 2013). Fungi in the genera *Alternaria*, *Colletotrichum*, *Cladosporium*, *Fusarium*, *Penicillium*, *Phytophthora*, and *Stagonosporopsis* have been reported to cause a range of leaf, fruit, stem, and/or root diseases in muskmelons (Li et al., 2015; Garampalli et al., 2016; Ghuffar et al., 2018). Gummy stem blight disease caused by *Stagonosporopsis cucurbitacearum* (synonym = *Phoma cucurbitacearum* and *Didymella bryoniae*), and wilt caused by *Fusarium oxysporum* f. sp. *melonis*, are important diseases associated with muskmelons and have been known to cause significant losses in yield and quality of this fruit (Gomez and Tello, 2000; Perchepied and Pitrat, 2004; Li et al., 2015; Garampalli et al., 2016). Other diseases that may pose a serious problem in muskmelons are anthracnose, powdery mildew, downy mildew, and necrotic spot virus (Kishi, 1966; Kristkova et al., 2009). Chemical fungicides are commonly used for the control of fungal pathogens. However, these chemical fungicides are known to be hazardous to farmers’ and consumers’ health, and environment (Lo, 2010; Yang, 2011). Many researchers have been interested in application of beneficial microorganisms, especially biological control agents in replacing chemical fungicides that can support the sustainability of the agriculture, produce safe food, and reduce environmental pollution (Cal et al., 2009; Wackett, 2013; Gava and Pinto, 2016).

Endophytic fungi are characterized by their ability to asymptomatically colonize in tissues of plants (Bacon and White, 2000; Rodriguez et al., 2009). Endophytic fungi benefit the host plant by enhancing growth, development, adaption, and stress tolerance (Carroll, 1988; Bacon and White, 2000; Rodriguez et al., 2009; Padhi et al., 2015). Interestingly, endophytic fungi have been shown to benefit their host by conferring protection against certain diseases resulting in reduced levels of infection, as well as suppression of the plant’s growth and/or a reduction in the amount of pathogens (Bacon and White, 2000; Gao et al., 2010; Selim et al., 2012). Several endophytic fungi, such as the *Cladosporium*, *Colletotrichum*, *Fusarium*, *Penicillium*, *Pestalotiopsis*, and *Trichoderma* species, have been used as attractive options for the management of some plant diseases (Bacon and White, 2000; Gao et al., 2010; Gava and Pinto, 2016). During our investigation of endophytic fungi in the Siam weed [*Chromolaena odorata* (L.) R. M. King & H. Rob.], we found *Trichoderma* to be a species of particular interest. Consequently, we have described this new species as *Trichoderma phayaoense* in this present paper. The abilities of this fungus as a potential biological agent to control *S*. *cucurbitacearum* and *Fusarium equiseti* has been noted in both *in vitro* and *in vivo* experiments. These pathogens are known to cause devastating gummy stem blight diseases and wilt in muskmelons, respectively. The *in vivo* potential of this fungus on plant growth was evaluated. Moreover, the ability of *T. phayaoense* to tolerate some commercial forms of fungicides, pesticides, and herbicides in the solid culture was investigated. The finding from this research study will be used to develop *T. phayaoense* as a biocontrol agent, which may then be used to replace the chemical fungicides that are currently being used.

## Materials and Methods

### Isolation of Endophytic Fungi

Endophytic fungi were isolated from the healthy leaves of Siam weed (*C. odorata*) collected from a native forest located on the campus of the University of Phayao, Phayao Province, Northern Thailand (19°1'42.6'' N, 99°53'38.9'' E) in May of 2015. Leaves were washed in running tap water for 15 min. The isolation of endophytic fungi was followed the method described by Nuangmek et al. (2008) with some modifications. The samples were then cut into small pieces (5 mm × 5 mm), then surface-disinfected by soaking in 75% ethanol for 30 s, 2% sodium hypochlorite solution for 3 min and 95% ethanol for 30 s. The sterilized samples were placed on potato dextrose agar (PDA; CONDA®, Spain) supplemented with 0.05% streptomycin sulfate and 0.03% rose Bengal in Petri plates. The isolation plates were incubated at 25°C in darkness until fungal growth was initiated. The tips of the fungal hyphae were aseptically removed and transferred to PDA. Each pure fungal isolate was kept on PDA slants for future use.

### Fungal Pathogens

Two fungal pathogens, *S. cucurbitacearum* SDBR-CMU292 (Nuangmek et al., 2018) and *F. equiseti* SDBR-UP-PA002 (Nuangmek et al., 2019), were used in this study. Both fungal strains were obtained from the Sustainable Development of Biological Resources (SDBR) Laboratory, Faculty of Science, Chiang Mai University. Fungal cultures were cultivated on PDA plate and grown in darkness for 1 week at 25°C.

### *In vitro* Test of Isolated Endophytic Fungi Against Mycelial Growth of *S. cucurbitacearum* and *F. equiseti*

Dual culture assay was used to test for inhibitory effects of the 21 isolates of endophytic fungi against *F. equiseti* SDBR-UP-PA002 and *S. cucurbitacearum* SDBR-CMU292 following the method described by Arnold et al. (2000). An agar plug (5 mm in diameter) of each endophytic fungus was cut from a 5-day-old colony on a PDA and was placed on one side of that PDA plate. A mycelial plug of pathogen was cut from a 5-day-old colony on PDA and was placed on the opposing side of the PDA plate. Plates were sealed with Parafilm M® (Sigma-Aldrich, United States) and then incubated at 25°C for 5 days. A mycelial plug of the pathogen was placed alone in the same manner on PDA plates serve as a control. The radial growth of pathogen colonies was measured. The percentage inhibition was calculated as has been previously described by Nuangmek et al. (2008) according to the following formula: [(*A*−*B*)/*A*] × 100 where *A* represents the radial growth of pathogens in the control and *B* represents the radial growth of pathogens after treatment. Three replicates were completed for each treatment, and experiments were repeated twice. The effective fungal isolate against the mycelial growth of both fungal pathogens was selected and used in future experiments.

### Identification of Selected Endophytic Fungus

#### Morphological Studies

The morphological characteristics were used to initially identify the selected endophytic fungus. Colony characteristics e.g., mycelial density, mycelial texture, and pigment production on different were recorded (Shah et al., 2012; Chaverri et al., 2015; Jaklitsch and Voglmayr, 2015; Chen and Zhuang, 2017). Furthermore, a light compound microscope (Olympus CX51, Japan) was used to examine the micromorphological characteristics. Size, shape, and structure data associated with the relevant anatomical features were measured at least 50 measurements of each structure.

#### Molecular Studies

The identification of a selected fungus was confirmed by molecular phylogenetic analysis. Genomic DNA was extracted from fungal cultured on PDA for 3 days, using FavoPrep® DNA Extraction Mini Kit (Taiwan). The internal transcribed spacers (ITS) was amplified with primers ITS4/ITS5 (White et al., 1990), the second largest subunit of RNA polymerase II (*rbp2*) genes was amplified by primers fRPB2-5f/fRPB2-7cr (Lui et al., 1999) and the translation elongation factor 1-alpha (*tef-1*) gene was amplified using primers EF1-728F/EF1-986R (Carbone and Kohn, 1999). The amplification program was conducted by running for 35 cycles for all gene regions. The initial denaturation at 95°C for 5 min, denaturation at 95°C for 30 s, annealing at 52°C for 30 s (ITS), 54°C for 45 s (*rbp2*), 52°C for 1 min (*tef-1*), extension at 72°C for 1 min, and a final cycle at 72°C for 10 min. The amplified PCR products were then checked by electrophoresis on 1% agarose gel stained with ethidium bromide and detected under UV light. The products were purified using NucleoSpin Gel and PCR Clean-up Kit (Macherey-Nagel, Germany), and then sent to a commercial sequencing provider (1^ST^ BASE Company, Kembangan, Malaysia). The obtained sequences were subjected to BLASTn search in GenBank.^1^

For phylogenetic analysis, the sequences obtained from this study and from previous studies, along with the sequences obtained from the GenBank database, were used (Supplementary Table S1). Multiple sequence alignment was carried out using MUSCLE (Edgar, 2004). The finalized alignment of a combined ITS, *rbp2*, and *tef-1* alignments in this study were submitted to TreeBASE under the study ID 27012. *Trichoderma ceramicum* and *T. parestonicum* were used as the outgroup. A phylogenetic tree was constructed using maximum likelihood (ML) and Bayesian inference (BI) methods. The best substitution models for ML and BI analyses were estimated by Akaike Information Criterion (AIC) in jModeltest 2.1.10 (Darriba et al., 2012). ML analysis was carried out on RAxML v7.0.3 under the GTR+I+G model with 1,000 bootstrap replications (Stamatakis, 2006). BI analysis was conducted with MrBayes v3.2.6 (Ronquist et al., 2012) to evaluate the posterior probabilities (PP) by Markov chain Monte Carlo sampling (MCMC). Markov chains were run for 1 million generations and trees were sampled every 100th generation and 10,000 trees were obtained. The first 2,000 trees representing the burning phase of the analyses were discarded. The remaining 8,000 trees were then used to calculate PP in the majority rule consensus tree. Bootstrap support (BS) and PP values greater than or equal to 70% and 0.95, respectively, of each branch were considered to be significantly supported (Felsenstein, 1985; Alfaro et al., 2003). The phylogenetic species were delimited in this study according to the genealogical concordance phylogenetic species recognition (GCPSR) criterion (Taylor et al., 2000). Using this method, phylogenetic species were recognized as genealogically exclusive under GCPSR if they were concordantly supported by multiple independent loci.

### Pathogenicity Test

For the pathogenicity test, selected endophytic fungus, *S. cucurbitacearum* SDBR-CMU292 and *F. equiseti* SDBR-UP-PA002, were evaluated for their ability to act as causal agents of diseases in muskmelons. Seeds of muskmelons (*C. melo* var. *inodorus*) were soaked in sterilized distilled water overnight. Commercial soil (Sunantha Nursery Company, Thailand) was used with a pH value of 6.5–6.8. The soil was sterilized at 121°C for 60 min. After cooling for 24 h, the sterilized soil was added into each 7 cm × 7.5 cm pot. Seeds were then placed in each pot. The experiment was conducted in a greenhouse (30 ± 2°C) located at the Faculty of Agriculture and Natural Resources, University of Phayao from February to March in 2018. The temperature, relative humidity, and maximum daily-light intensity ranged from 30 to 34°C, 60 to 75%, and 13,500 to 57,000 lux, respectively. Water was added every other day. After 2 weeks, seedlings were transferred into each 14 cm × 19 cm pot containing sterilized soil. Seedlings were then continuously grown for 1 week and used in the pathogenicity test. A conidial suspension of each fungus was prepared following the method described by Li and Brewer (2016). Fungal cultured on PDA for 2 weeks was flooded with a sterile 0.05% (v/v) Tween 80 and 0.85% (w/v) NaCl aqueous solution. The conidial suspension was filtrated through two layers of sterilized cheesecloth. The conidia concentration was adjusted by using a hemocytometer to a final concentration of approximately 1 × 10^6^ conidia/ml. Inoculation was done by addition of 10 ml conidial suspension onto the soil surface around the base of the seedlings. Seedlings treated with sterile distilled water served as control. Disease symptoms were observed. Ten replicates per each treatment were generated. All treatments were repeated twice. The re-isolation of fungi from any lesions that developed on inoculated plants was performed by the single spore isolation method as described by Choi et al. (1999) in order to complete Koch’s postulates.

### Plant Growth Promotion and Control of Gummy Stem Blight and Wilt in Muskmelon With a Selected Endophytic Fungus in Greenhouse

#### Seedlings

Seedlings of muskmelons were prepared following the method described above. One two-week-old seedling was transferred into each plastic bag (20.32 cm × 33.02 cm) containing sterilized commercial soil. Seedlings were continuously grown for 2 weeks and were then used in this experiment.

#### Fungal Inoculum Preparation

A selected endophytic fungus and fungal pathogens (*S. cucurbitacearum* SDBR-CMU292 and *F. equiseti* SDBR-UP-PA002) were cultivated on PDA. A conidial suspension of each fungus was prepared following the method described above. The final conidial concentration was adjusted to 1 × 10^6^ conidia/ml before being used.

#### Experimental Design

Six treatments were tested to establish the effectiveness of the selected endophytic fungus on controlling gummy stem blight and wilt in muskmelons under greenhouse conditions (Supplementary Table S2). Fifteen milliliters of conidial suspension of a selected endophytic fungus were poured onto soil surface around seedlings. Sterile distilled water was used as a control. After 1 week of inoculation with a selected endophytic fungus, 10 ml of conidial suspension of each pathogenic fungus was added onto soil surface around seedlings. All treatments were conducted in a greenhouse from March to May in 2018. The temperature, relative humidity, and maximum daily-light intensity ranged from 32 to 36°C, 60 to 70%, and 14,500 to 59,000 lux, respectively. Water was added every other day. The symptoms of gummy stem blight and wilt were observed after 5 weeks of incubation. The disease severity index (DSI) for each pathogen was evaluated. For *F. equiseti*, the DSI was applied by following the description of Boughalleb et al. (2007) with some modifications to the scales: 0 = healthy; 1 = slight yellowing of leaves with slight rot pivot and lateral roots and crown rot; 2 = significant yellowing in leaves with or without wilting, stunting of plants, severe rot at the pivot and lateral roots, significant rot, and browning of vessels in the stem; and 3 = death of the plant. The DSI of *S. cucurbitacearum* was applied followed the method described by Dalcin et al. (2017) with slight modifications to the scales: 0 = healthy; 1 = plants with less than 1% of damaged leaf area; 2 = plants between 1 and 5% of damaged leaf area; 3 = plants between 6 and 25% damaged leaf area; 4 = pants between 26 and 50% of damaged leaf area; 5 = plants showing more than 50% of damaged leaf area. An experiment was conducted as a completely randomized design (CRD) with 10 replications each. All treatments were repeated twice.

#### Measurement of Plant Growth and Fruit Production

Plant height, the number of leaves, the dry weight of the shoot (including the leaves) and the dry weight of the roots were recorded during incubation periods. After 5 weeks of incubation, the fruit of the plants reached the maturity stage for harvesting. In this study, the number of fruits for all plants was set at one fruit per plant. Fruit weight, diameter of the fruit, circumference of the fruit, and fruit quality parameters (total soluble solid, total titratable acidity, and fruit firmness values) were measured.

### Fungicide, Herbicide, and Insecticide Tolerance of Selected Endophytic Fungus

Three commercial fungicides, metalaxyl (Metalaxyl 35 W®, Thailand), pyraclostrobin (Headline®, Thailand), and copper hydroxide (Funguran-OH®, Thailand), one insecticide, carbamate (Carbaryl 85®, Thailand), and one herbicide, paraquat dichloride (Grammoxone®, Thailand) were tested in this experiment. The aqueous stock solution of metalaxyl, pyraclostrobin, copper hydroxide, carbamate, and paraquat dichloride were prepared at 35000, 55000, 75,000, 85,000, and 1,000 ppm, respectively, according the method described by Suwannarach et al. (2015). The recommended field dosages of metalaxyl, pyraclostrobin, copper hydroxide, carbamate, and paraquat dichloride were 500, 500, 1,000, 1,000, and 6.25 ppm, respectively. The aqueous stock solution of each compound added to a sterilize PDA at final concentrations of half recommended dosages to quintuple the recommended dosage. The surface of the test media was covered by a sterile cellophane disc, and the mycelial plug (5 mm in diameter) of the selected endophytic fungus was placed onto the test media. The plates were incubated at 25°C in darkness. After 2 weeks of incubation, the cellophane disc was removed and dried at 60°C for 48 h. Then, mycelium dry weights were measured. The tolerance index (TI) was calculated following the formula described by Fomina et al. (2005). TI values at 0% indicated a lethal effect and lower than 50% indicated a growth inhibition effect. Four replicates were made for each treatment.

### Statistical Analysis

The data were analyzed by one-way analysis of variance (ANOVA) using SPSS program version 16.0 for Windows. The significant differences (*p* ≤ 0.05) between the mean values of each treatment were determined using Duncan’s multiple range test.

## Results

### Inhibitory Effects of Endophytic Fungi Against Fungal Pathogens

**Figure 1:**
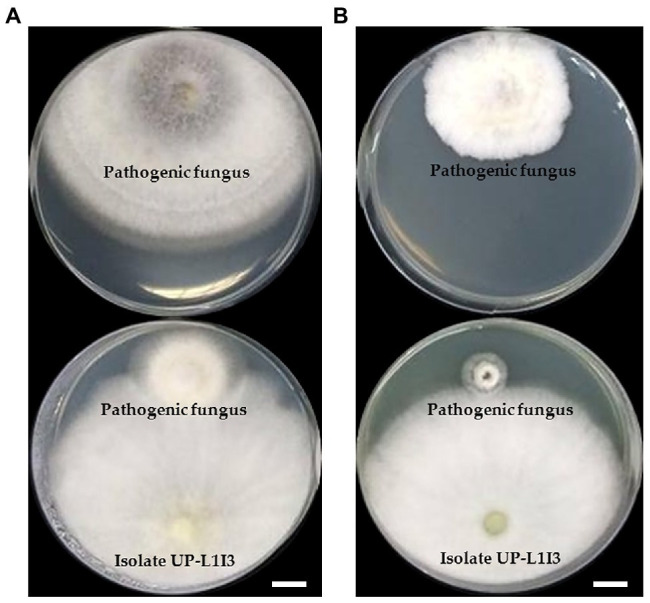
Dual culture assay of an endophytic fungus isolate UP-L1I3 against mycelia growth of *Fusarium equiseti* **(A)** and *Stagonosporopsis cucurbitacearum* **(B)** on potato dextrose agar (PDA) after 5 day of incubation at 25°C in darkness. The upper plate was a control plate, and the lower pate was an experimental plate. Scale bars = 10 mm.

Twenty-one fungal isolates were recovered from the leaves of the Siam weed. The results showed that the fungal isolate UP-L1I3 showed the highest degree of percentage for the inhibition of mycelial growth of *F. equiseti* SDBR-UP-PA002 and *S. cucurbitacearum* SDBR-CMU292 at 90.80 and 81.60%, respectively, by dual culture assay (Table 1; Figure 1). This observation was informed by measurements of growth. An overgrowth of fungal isolate UP-L1I3 on both fungal pathogens was observed. It could be explained that the inhibition effect was parasitism. Therefore, the isolate UP-L1I3 was selected and used for future experiments. This fungal isolate was deposited at SDBR Laboratory and Thailand Bioresource Research Center (TBRC) under number SDBR-CMU349 and TBRC10852, respectively.

**Table 1:** Mycelial growth inhibition of 21 fungal isolates against *F. equiseti* and *S. cucurbitacearum* assessed by dual culture assay.

Fungal isolate	Mycelial growth inhibition (%)^*^	
	*Fusarium equiseti*	*Stagonosporopsis cucurbitacearum*
UP-PY01	50.00 ± 0.50^n^	35.25 ± 0.90^t^
UP-EX1R1	81.37 ± 0.30^c^	59.71 ± 0.49^i^
UP-EX1R2	80.56 ± 1.23^cd^	57.51 ± 0.63^j^
UP-PY02	75.97 ± 0.18^gh^	53.80 ± 0.60^k^
UP-EX1R3	73.49 ± 1.01^ij^	64.10 ± 0.20^h^
UP-PY03	57.81 ± 1.21^m^	39.37 ± 1.15^s^
UP-EX3R1	82.33 ± 0.50^c^	69.96 ± 0.45^d^
UP-EX4R2	62.30 ± 0.25^l^	45.72 ± 0.74^r^
UP-EX2R3	79.11 ± 1.19^de^	49.54 ± 0.51^n^
UP-EX2R1	72.43 ± 0.54^j^	53.28 ± 0.41^l^
UP-PY05	51.56 ± 0.44^n^	29.22 ± 1.24^u^
UP-EX2R2	78.26 ± 1.91^ef^	51.69 ± 0.31^m^
UP-PY14	77.03 ± 1.52^efg^	64.70 ± 1.30^f^
UP-EX3R3	75.50 ± 0.56^fgh^	63.21 ± 0.23^g^
UP-EX3R2	69.76 ± 1.31^k^	49.15 ± 0.60^o^
UP-L1I3	90.80 ± 1.89^a^	81.60 ± 2.83^a^
UP-L27I2	84.45 ± 2.49^b^	78.33 ± 0.94^c^
UP-R24I2	84.06 ± 1.26^b^	79.00 ± 1.10^b^
UP-EX4R2	61.60 ± 1.10^l^	47.80 ± 0.95^p^
UP-EX4R1	58.80 ± 1.30^m^	46.18 ± 0.20^q^
UP-EX3R3	74.73 ± 0.93^hi^	66.96 ± 0.97^e^

The different letters in the same column are significantly different (*p* < 0.05) according to Duncan’s multiple range test.

* The results are mean of three replicates ± standard deviations (SD).

### Identification of Selected Endophytic Fungus

#### Morphological Observation

Fungal colonies of isolate UP-L1I3 on corn meal dextrose agar (CMD), PDA, and synthetic nutrient-poor agar (SDN) were reached 32–33, 30–34, and 30–32 mm in diameter at 25°C for 3 days, respectively. The conidia production was observed in all agar media. The fungal isolate UP-L1I3 produced conidiophores often on long main axis, ampulliform to lageniform phialie (3.2–3.3 length/wide ratio), smooth globose to subglobose with rarely broadly ellipsoid conidia and chlamydospores. The fungal isolate UP-L1I3 was initially identified as belonging to the *Trichoderma harzianum* species complex based on the morphological observations (Gams and Meyer, 1998; Samuels, 2006; Chaverri et al., 2015). Therefore, molecular methods were applied to confirm the identification of this fungal isolate.

#### Phylogenetic Results

**Figure 2:**
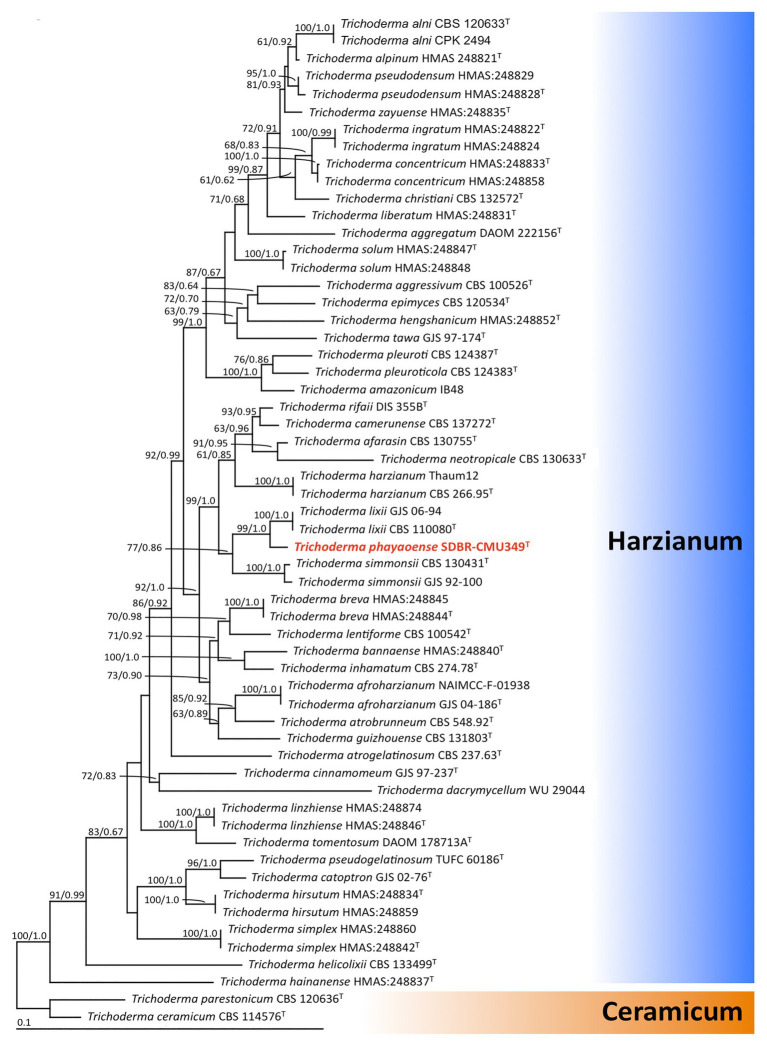
The phylogenic tree obtained by maximum likelihood analysis of the combined ITS, *rpb2*, and *tef-1* genes of 58 specimens of *Trichoderma*. *Trichoderma ceramicum*, and *Trichoderma parestonicum* were used as an outgroup. The set of numbers above the nodes are BS value (left) and PP value (right) expressed values above 50% and 0.95, respectively. Superscription “T” means the type species and the fungal species obtained in this study is in red.

The sequences of ITS, *rbp2*, and *tef-1* obtained from fungal isolate UP-L1I3 (SDBR-CMU349) were deposited in GenBank database under the accession numbers MT995122, MW002073, and MW002074, respectively (Supplementary Table S1). The combined ITS, *rpb2*, and *tef-1* sequence dataset consisted of 58 taxa, while the aligned dataset was comprised of 3,031 characters including gaps (ITS: 1–608, *rpb2*: 609–1,693, and *tef-1*: 1694–3,031). The average SD of the split frequencies of BI analysis was 0.030270. ML analysis revealed the proportion of invariable sites and a gamma distribution with shape parameters for the rates of nucleotide substitution among the variable sites at 0.7570 and 0.5560, respectively. The tree with a final log likelihood value of −14876.59 was obtained. The phylogram of ML and BI analyses were similar in topology. Therefore, we have only presented the phylogram obtained from ML analysis (Figure 2). Our phylogenetic tree was constructed concordantly and was supported by previous studies (Chaverri et al., 2015; Jaklitsch and Voglmayr, 2015; Chen and Zhuang, 2017). A phylogram assigned the fungal isolate UP-L1I3 as a new species which described herein as *T. phayaoense* into the Harzianum calde. *Trichoderma phayaoense* was clearly distinguished from other *Trichoderma* species in the Harzianum clade and formed a sister taxon to *Trichoderma lixii* with high bootstrap (99%) and posterior probability (1.0) supports. According to the genealogical concordance phylogenetic species recognition criterion of Taylor et al. (2000), *T. phayaoense*, in this study, should be recognized as belonging to an independent phylogenetic species.

#### Taxonomic Description

**Figure 3:**
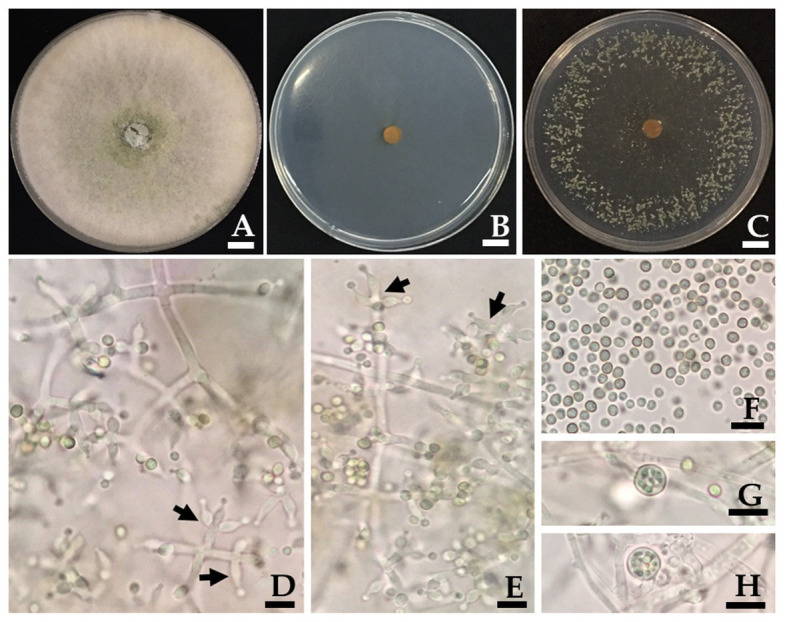
*Trichoderma phayaoense* SDBR-CMU349 (holotype). Colony on PDA **(A)**, corn meal dextrose agar (CMD; **B**) and SNA **(C)** after 5 days of incubation at 25°C in darkness. **(D,E)** Phialide (arrows point), **(F)** Conidia, and **(G,H)** Chlamydospores. Scale bars **(A–C)** = 10 mm, **(D–H)** = 10 μm.

*Trichoderma phayaoense* W. Nuangmek & N. Suwannarach sp. nov. Figure 3.

MycoBank no.: 837510.

Etymology: “*phayaoense*” referring to Phayao Province, Thailand, which the new fungus was isolated from *C. odorata*.

Holotype: THAILAND. Phayao Province, Mueang District, (19°1'42.6''N, 99°53'38.9508''E), isolated from *C. odorata* in deciduous dipterocarp forest of The University of Phayao, May 2015, W. Nuangmek, dried cultures: SDBR-CMU349; ex-type living culture: TBRC10852.

Gene sequences: MT995122 (ITS), MW002073 (*rpb2*), and MW002074 (*tef-1*).

Culture characteristics: Fungal colonies on PDA, CMD and SDN reached 30–34, 30–32, and 32–33 mm in diameter at 25°C for 3 days, respectively, while mycelium covered the plates after 5 days of incubation. Colonies on PDA were circular, dense, aerial hyphae abundant but loosely disposed, forming a white mat, becoming floccose to granular in the center and turning pale yellowish green (Figure 3A). Colonies on CMD were circular, flat, homogeneous, and mycelium loose (Figure 3B). Colonies on SNA were circular but not dense, surface becoming downy due to numerous long aerial hyphae except in the center (Figure 3C). No distinct odor, while no diffusing pigment was observed in all agar media. Conidiation began after 3 days in all agar media and were numerous and effused on aerial hyphae. Conidiophores symmetrical, trichoderma-like, often with a long main axis up to 150 μm, side branches short with no rebranching. Phialides formed solitary, paired or in whorls of 3, ampulliform to lageniform, 6.7–9.8 × 2.0–3.0 μm, 3.2–3.3 length/wide ratio, 1.0–1.5 μm wide at the base (*n* = 50; Figures 3D,E). Conidia green, smooth, globose to subglobose, rarely broadly ellipsoid, 2.0–3.9 × 2.0–2.9 μm (*n* = 50), and 1.0–1.3 length/wide ratio (Figures 3D–F). Chlamydospores were only observed in PDA and were terminal and intercalary of hyphae, globose, or pyriform 5.9–7.8 × 4.9–7.8 μm (*n* = 50; Figures 3G,H).

Habitat: Isolated from leaves of *C. odorata* in deciduous dipterocarp forest.

Geographic distribution: Know only from Thailand.

Morphologically, *T. phayaoense* is characterized by its developed conidiophores and its often long main axis. Furthermore, it possesses ampulliform to lageniform phialie with 3.2–3.3 length/width ratio. It is characterized as smooth globose to subglobose with rarely broadly ellipsoid conidia and the presence of chlamydospores. Thus, these morphological characteristics support its placement within the *T. harzianum* complex (Gams and Meyer, 1998; Samuels, 2006; Chaverri et al., 2015). The characteristics of the colonies of *T. phayaoense* are similar to those of *Trichoderma afarasin*, *T. harzianum*, *Trichoderma lentiforme*, *T. lixii*, and *Trichoderma simmonsii*. The size of the conidia and phaialde, and the degrees of chlamydospore production and geographic distribution of *T. phayaoense*, were also compared to other related *Trichoderma* species (Table 2). Chlamydospores were observed in cultures of *T. phayaoense* and were similar to those of *T. afarasin*, *T. harzianum*, and *T. simmonsii* (Blanchette, 1991; Shah et al., 2012; Chaverri et al., 2015). However, the width of the phialide of *T. phayaoense* was narrower than that of other related species. The phialide length of *T. afarasin* (5.3–6.0 μm), *T. lentiforme* (5.3–5.5 μm), *T. lixii* (5.3–6.0 μm), and *T. simmonsii* (6.1–6.5 μm) was distinguishable from our new species (6.7–9.8 μm; Chaverri et al., 2015). Moreover, the width of conidia of *T. phayaoense* (2.0–2.9 μm) was found to be narrower than *T. lixii* (3.1–3.2 μm) and *T. simmonsii* (3.0–3.1 μm wide; Chaverri et al., 2015). Multi-locus phylogenetic analysis confirmed that *T. phayaoense* clearly separated it from other *Trichoderma* species within the Harzianum calde. Consequently, it forms a sister taxon to *T. lixii* (Figure 2). *Trichoderma lixii* differs from *T. phayaoense* by the absence of chlamydospores and the different phialide and conidia sizes of *T. phayaoense* and *T. lixii* that have been mentioned above (Chaverri et al., 2015).

**Table 2:** Distribution, chlamydospores observation, and microscopic observation of *Trichoderma phayaoense* with the closely related species.

*Trichoderma* species	Distribution	Chlamydospores observation	Microscopic observation			
			Conidia		Phialide	
			Length (μm)	Width (μm)	Length (μm)	Width (μm)
*T. afarasin*^a^	West Africa	+	2.8 (2.7–2.9)	2.6 (2.5–2.6)	5.6 (5.3–6.0)	3.2 (3.1–3.4)
*T*. *harzianum*^a^^,^^b^^,^^c^	Africa and Asia, Europe, North America, Oceania, and South America	+	3.2 (3.1–3.2)	2.8 (2.7–2.8)	6.9 (6.7–7.2)	3.5 (3.5–3.6)
*T. lentiforme*^a^	Neotropic	−	2.8 (2.8–3.0)	2.6 (2.5–2.7)	5.4 (5.3–5.5)	3.5 (3.5–3.52)
*T. lixii*^a^	Southeast Asia	−	3.2 (3.1–3.2)	3.2 (3.1–3.2)	6.7 (6.3–7.0)	3.7 (3.6–3.8)
*T. simmonsii*^a^	Europe and North America	+	3.0 (3.0–3.1)	3.0 (3.0–3.1)	6.3 (6.1–6.5)	3.3 (3.3–3.4)
*T. phayaoense*^d^	Thailand	+	3.0 (2.0–3.9)	2.5 (2.0–2.9)	8.1 (6.7–9.8)	2.7 (2.0–3.0)

“+” = presence and “−” = absence.

a Chaverri et al. (2015).

b Blanchette (1991).

c Shah et al. (2012).

d This study.

### Pathogenicity Test

It was found that no disease symptoms were observed on the seedlings inoculated with *T. phayaoense* and the control seedlings. Therefore, *T. phayaoense* was determined to be non-pathogenic to the muskmelon plant. However, disease symptoms of gummy stem blight developed on the inoculated seedlings after 2 weeks of incubation. The initial symptoms of the gummy stem blight appeared on the stems by brown to dark brown spots and develop into elliptical- to oblong-shaped lesions. Subsequently, stem cankers developed in the cortical tissue, water soaked, and the presence of brown gummy exudates formed on the surface. Cankers expanded, while wilted leaves of the vines became blighted and desiccated. Additionally, initial symptoms of wilt were observed after 2 weeks of incubation, wherein leaves turned yellow or dull green and became brown and dry followed by the death of the lateral branches. A vascular brown discoloration appeared on the stem. *Fusarium equiseti* and *S. cucurbitacearum* were also isolated from the lesions of wilt and gummy stem blight, respectively.

### Plant Growth Promotion and Control of Gummy Stem Blight and Wilt in Muskmelon With *T. phayaoense*

**Figure 4:**
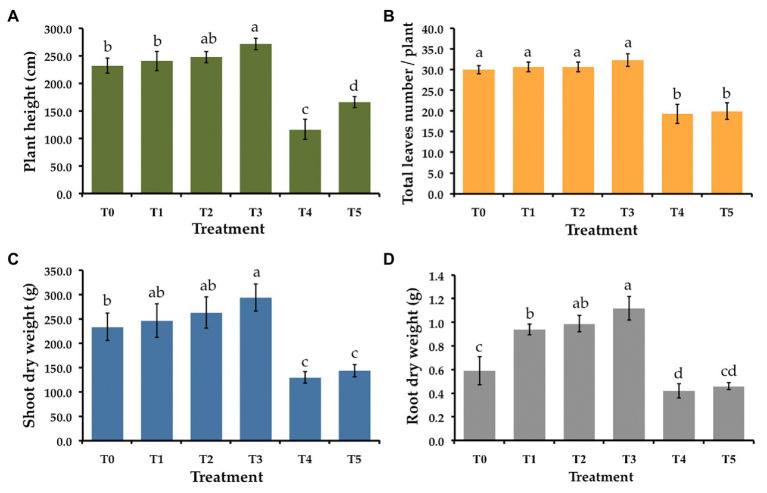
Effect of *T. phayaoense*, *F. equiseti*, and *S. cucurbitacearum* treatments on the growth of muskmelon plant. **(A)** Plant height, **(B)** total leaves number per plant, **(C)** shoot dry weight, and **(D)** root dry weight. Error bars represent standard deviation of the mean. Different letter indicate the significantly different at *p* ≤ 0.05.

Six treatments were tested to establish the effectiveness of the selected endophytic fungus on controlling gummy stem blight and Fusarium wilt in muskmelons under greenhouse conditions. The disease severity index (DSI) of each treatment is shown in Table 3. After 5 weeks in a greenhouse, both conidial inoculations of *F. equiseti* (T4) and *S. cucurbitacearum* (T5) were found to produce the highest DSI values. In contrast, there was no lesion development on the seedling control (T0) and on the seedlings treated with *T. phayaoense* (T3). Furthermore, *T. phayaoense* significantly decreased DSI values in terms of both wilt and gummy stem blight of the seedlings (T1 and T2). After 5 weeks, muskmelon seedlings from each experiment were measured for shoot height, the number of leaves, the dry weight of shoots, and the dry weight of roots. The shoot height, number of leaves, shoot dry weight, and root dry weight of the seedlings in *F. equiseti* (T4) and *S. cucurbitacearum* (T5) treatments were significantly lower than in all other treatments (Figure 4). It was found that the *T. phayaoense* treatment (T3) did increase shoot height, shoot dry weight, and root dry weight when compared to the control (T0). It was also found that muskmelon seedlings treated with *T. phayaoense* and inoculated with *F. equiseti* (T1) and *S. cucurbitacearum* (T2) displayed increases in shoot height, shoot dry weight, and root dry weight when compared to the control.

**Table 3:** The disease severity index (DSI) of muskmelon seedling of each treatment.

Treatment number	DSI value^*^	
	Wilt	Gummy stem blight
T0	0.00^c^	0.00^c^
T1	0.40 ± 0.52^b^	ND
T2	ND	2.00 ± 0.67^b^
T3	0.00^c^	0.00^c^
T4	1.90 ± 0.32^a^	ND
T5	ND	4.20 ± 0.79^a^

Values with the different letters in the same column are significantly different (*p <* 0.05) according to Duncan’s multiple range test.

* Data are means ± SD. “ND” = not determined.

**Figure 5:**
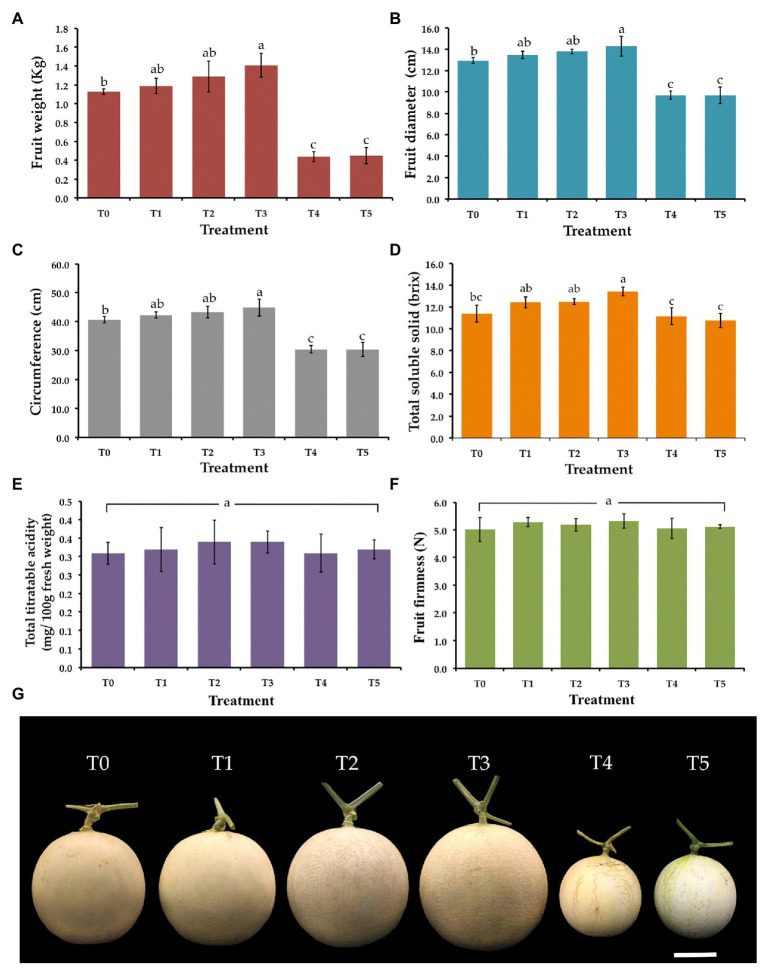
Effect of *T. phayaoense*, *F. equiseti*, and *S. cucurbitacearum* treatments on the fruit of muskmelon. **(A)** Fruit weight, **(B)** fruit diameter, **(C)** circumference, **(D)** total soluble solids, **(E)** total titrable acidity, **(F)** fruit firmness, and **(G)** muskmelon fruits in each treatment. Error bars represent SD of the mean. Error bars represent standard deviation of the mean. Different letter indicate the significantly different at *p* ≤ 0.05. Scale bar = 5 cm.

Weight, diameter, circumference, total solid soluble values, total titratable acidity, and fruit firmness of all muskmelon fruits in each treatment were measured and the data are shown in Figure 5. The value of weight, diameter, and circumference of fruits in both *F. equiseti* (T4) and *S. cucurbitacearum* (T5) treatments were significantly lower than in other treatments. The results indicated that the fruits receiving the *T. phayaoense* treatment (T3) displayed higher values in terms of weight, diameter, and circumference when compared to the control (T0). Additionally, the fruits of the seedlings treated with *T. phayaoense* and inoculated with *F. equiseti* (T1) and *S. cucurbitacearum* (T2) displayed higher values in terms of weight, diameter, and circumference when compared to the control (T0), but these values were not found to be significantly different. The result showed that the fruits receiving the *T. phayaoense* treatment (T3) have the highest value of total soluble solid. Notably, total titratable acidity and fruit firmness in all treatments were not found to be significantly different.

### Fungicide, Herbicide, and Insecticide Tolerance of *T. phayaoense*

**Figure 6:**
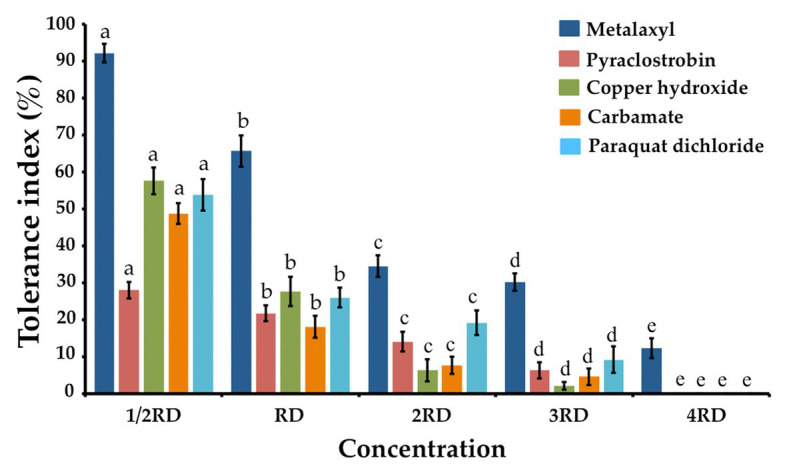
Tolerance fungicide, herbicides and insecticide index of *T. phayaoense*. Data were means of four replicates. Error bars represent SD of the mean. Different letter indicate the significantly different at *p* ≤ 0.05. 1/2 RD, RD, 2RD, 3RD, and 4RD indicate the half recommended dosage, recommended dosage, double recommended dosage, triple recommended dosage and quadruple recommended dosage, respectively.

The fungicide, herbicide, and insecticide tolerance capabilities of *T. phayaoense* were reported in terms of a TI value and are showed in Figure 6. It was found that the TI value decreased when the concentration of all chemical compounds increased. The result indicated that the growth of *T. phayaoense* was inhibited (TI value < 50%) by pyraclostrobin and carbamate in all tested dosages. It was found that this fungus was tolerant (IT value > 50%) in recommended dosages of metalaxyl and in half-recommended dosages of copper hydroxide and paraquat dichloride.

## Discussion

This study constitutes an investigation of the potential ability of endophytic fungi obtained from Siam weed leaves collected from Northern Thailand in the control of gummy stem blight and wilt diseases in muskmelon seedlings. The fungal isolate UP-L1I3 was selected and used based on the determination that this isolate displayed the highest percentage values for inhibition of the mycelial growth of *F. equiseti* and *S. cucurbitacearum*. The present study has identified the fungal isolate UP-L1I3 as a new endophytic fungal species of *Trichoderma*, which has been described herein as *T. phayaoense* based on a combination of its morphological characteristics and an extensive multilocus (*ITS*, *rbp2*, and *tef-1* genes) phylogenetic analysis (Chaverri et al., 2015; Jaklitsch and Voglmayr, 2015; Chen and Zhuang, 2017). Several previous studies have reported that the *Trichoderma* species have been found on a wide variety of substrates and have been isolated from rotting plant material, soil, and other organisms (Samuels, 2006; Jaklitsch, 2009; Jaklitsch and Voglmayr, 2015; Chen and Zhuang, 2017). Recently, it has been recognized as one of the most commonly isolated species of endophytes in many plants (Samuels, 2006; Jaklitsch, 2009; Chaverri and Samuels, 2013; Thapa et al., 2020). In this study, *T. phayaoense* could reduce the degree of mycelial growth of *S. cucurbitacearum* and *F. equiseti* in the dual culture assay through the parasitism effect. These results are similar to those of previous studies (Ru and Di, 2012; Sánchez-García et al., 2017; Chounhary and Ashraf, 2019; Haque et al., 2020) which reported that the *Trichoderma* species could inhibit the mycelial growth of several plant pathogenic fungi by the parasitism, antifungal production, and a competition for space and nutrients. Additionally, the inhibition efficiency was varied among *Trichoderma* species and strains, as well as being dependent upon the species of fungal plant pathogens (Hermosa et al., 2012; Ru and Di, 2012; Dawidziuk et al., 2016; Bunbury-Blanchette and Walker, 2019; Haque et al., 2020).

Many diseases that are caused by fungi can infect and damage of the melons plants (including cantaloupes and muskmelons) during the growing season, the harvesting process and storage, all of which can result in significant losses in the yield and quality of this fruit crop (Gomez and Tello, 2000; Walcott et al., 2004; Ghuffar et al., 2018; Li et al., 2015). Gummy stem blight disease caused by *S. cucurbitacearum* on a number of melon plants is distributed in many countries throughout the world (Li et al., 2015; Garampalli et al., 2016; Nuangmek et al., 2018). In the United States, gummy stem blight disease on cantaloupes caused by *S. citrull* has been previously reported (Stewart et al., 2015). In addition, Fusarium wilt is an important disease that is associated with muskmelons. Generally, *F. oxysporum* f. sp. *melonis* is known to be a causal agent of this disease (Punja et al., 2001; Perchepied and Pitrat, 2004; Garampalli et al., 2016). *Fusarium oxysporum* f. sp. *niveum* and *F. solani* have been reported as causal agents of melon wilt disease (Egel and Martyn, 2007; González et al., 2020). However, no incidence of wilt disease from *F. equiseti* had been reported in muskmelons. The symptoms of this disease caused by *F. equiseti* in muskmelons observed in the current study are similar to those that are known to be caused by *Fusarium* pathogens. Therefore, *F. equiseti* could be considered as one of the causal agents of wilt disease in muskmelons.

*Trichoderma* species have been widely known aspect of biological control agents that can serve as a multifaceted mechanism (in competition for nutrients and space, and mycoparasitism; Hyder et al., 2007; Schuster and Schmoll, 2010; Thapa et al., 2020). This study found that *T. phayaoense* could inhibit the mycelial growth of *S. cucurbitacearum* and *F. equiseti* and reduce the impact of the disease on the disease severity index for gummy stem blight and wilt disease in muskmelon seedlings. This determination is similar to the findings of other previous reports. Similarly, previous studies have determined that *Trichoderma* species (e.g., *T. akoningii*, *T. asperlloides*, *T. atroviride*, *T. harzianum, T. lentiforme, T*. *longibrachiatum*, and *T. virens*) successfully controlled various fungal plant pathogens including the fungal genera *Alternaria*, *Cladosporium*, *Fusarium*, *Phytophthora*, and *Stagonosporopsis* (Puyam, 2016; Ghazanfar et al., 2018; Sood et al., 2020; Thapa et al., 2020). Furthermore, Utkhede and Koch (2004) and Patel et al. (2017) found that *T. harzanum* could significantly reduce the effects of gummy stem blight disease caused by *S. cucurbitacearum* in cucurbit plants under both greenhouse and field conditions. Moreover, Gava and Pinto (2016) found that *T. harzianum*, *T. koningii*, *T*. *polysporum*, and *T. viride* showed a degree of efficiency in the control of melon wilt disease caused by *F. oxysporum* f. sp. *melonis*, which could then lead to increases in fruit production. A study conducted by Martínez-Medina et al. (2014) on *T. harzianum*, *T. ghanense*, and *T. hamatum* reported on their antagonistic activity to *F. oxysporum* f. sp. *melonis in vitro*, and their biocontrol activity against Fusarium wilt on melon plants in field experiments. In this present study, the inoculation of *T. phayaoense* could be effective in improving plant development (increasing plant height and dry weight values of shoots and roots) and the number of fruit attributes (weight, diameter, circumference, and total soluble solids) of muskmelons. These results were similar to those of Fernondo et al. (2018) who found that *T. saturnisporum* enhanced the growth of melon seedlings, as well as increased fruit productivity and quality when compared with the control. Moreover, previous studies have reported that *Trichoderma* spp. could promote the growth of bitter gourds, bottle gourds, cucumbers, peppers, rice, and tomato seedlings (Chagas et al., 2015; Kotasthane et al., 2015; Marin-Guirao et al., 2016; Herrera-Parra et al., 2017; Uddin et al., 2018). Additionally, *Trichoderma* spp. can produce phytohormones [indole-3-acetic acid (IAA) or auxin analogues, abscisic acid, and gibberellin], siderophores, and other plant growth promoting substances (e.g., 6-pentyl-α-pyrone, cyclonerodiol, harzianolide, and koninginins; Contreras-Cornejo et al., 2009; Vinale et al., 2012; Martínez-Medina et al., 2014; Zhao and Zhang, 2015; Zúñiga-Silgado and Vargas, 2016; Jaroszuk-Ściseł et al., 2019). Moreover, several previous studies have shown that *Trichoderma* spp. can solubilize several plant nutrients, especially phosphate, into an available form for plant that can improve plant growth (Chagas et al., 2015; Zhao and Zhang, 2015; França et al., 2017; Bononi et al., 2020).

The degrees of tolerance to fungicide, herbicide, and insecticide microorganisms indicate the capability of this species to effectively degrade those fungicides, herbicides, and insecticides. It was also found that tolerance activities varied in different plants depending on the types of compounds being used as well as the fungal isolates being employed (Ortiz-Hernández and Sánchez-Salinas, 2010; Chaparro et al., 2011; Singh et al., 2012; Mohiddin and Khan, 2013). An increase in the concentrations of chemical compounds decreased both fungal growth and TI values, which was in accordance with previous findings (Suwannarach et al., 2015; Boat et al., 2018; Karaoglu et al., 2018). In this study, *T. phayaoense* could tolerate the selected fungicide (metalaxyl) in the field at recommended dosages. Similar to our results, previous studies have shown that *T. atroviride, T. harzianum*, *T. koningii*, and *T. pseudokoningii* have been acknowledged for their ability to tolerate fungicides, herbicides, and insecticides (Khan and Shahzad, 2007; Chaparro et al., 2011; Singh et al., 2012; Bhale and Rajkonda, 2015; da Silva et al., 2018). Additionally, the biocontrol agents of *T. harzianum* and *T. virens* could tolerate different insecticide compounds and could display varying degrees of tolerance (Wedajo, 2015). Therefore, information on the effects of fungicides, herbicides, and insecticides on biocontrol agents would be beneficial to a range of important *in vivo* applications.

## Conclusion

Many researchers have been an increased interest in application of beneficial microorganisms for the sustainable agriculture in last decade. In this study, a new endophytic fungus, *T. phayaoense*, was isolated from the leaves of the Siam weed. Findings indicated the presence of the controlling muskmelon pathogens, *F. equiseti* and *S. cucurbitacearum*, in an *in vitro* investigation. A pathogenicity test confirmed that *T. phayaoense* could serve as a non-pathogen on muskmelon seedlings. Furthermore, *T. phayaoense* could prevent gummy stem blight and Fusarium wilt in muskmelon seedlings and could also be effective in improving plant and fruit development. Moreover, this fungus was found to be able to tolerate metalaxyl in field applications at recommended dosages. Thus, it is possible that *T. phayaoense* could be used as a plant growth promotion and biological control agent to manage gummy stem blight and wilt in muskmelons. It could also be demonstrated that the use of chemical fungicides can be replaced by this fungus. Further studies, the optimal techniques of inoculum production will be investigated, and its ability to produce chitinase, β-glucosidase, β-N-acetyl-D-hexosaminidase, and plant growth promotion substances will be determined. The toxicity assays are also necessary in laboratory, and clinical tests are needed in future studies to fully understand the profile of this fungus. Additionally, field trials will need to be conducted in the future.

## Data Availability Statement

The original contributions presented in the study are publicly available. This data can be found in NCBI’s GenBank with accession numbers: MT995122, MW002073, and MW002074.

## Author Contributions

WN, WA, and NS: conceptualization. WA, NS, and JK: methodology, software, formal analysis, investigation, data curation, and writing-original draft. WN, WA, NS, and SL: validation. WN and WA: resources. WN, WA, JK, NS, and SL: writing-review and editing. SL: supervision. All authors read, revised, and approved the final manuscript.

### Conflict of Interest

The authors declare that the research was conducted in the absence of any commercial or financial relationships that could be construed as a potential conflict of interest.

## Footnotes

^1^http://blast.ddbj.nig.ac.jp/top-e.html

## Supplementary Material

Supplementary Table S1 Details of sequences used in molecular phylogenetic analysis.

Supplementary Table S2 Treatment details in this study.
