# Supplementary material for: Evaluation of a Newly Identified Endophytic Fungus, Trichoderma phayaoense for Plant Growth Promotion and Biological Control of Gummy Stem Blight and Wilt of Muskmelon
Source: Front Microbiol. 2021 Mar 5;12:634772. doi: 10.3389/fmicb.2021.634772 (PMC7973005; doi:10.3389/fmicb.2021.634772)
Supplement: Supplementary Table S1 — Details of sequences used in molecular phylogenetic analysis. [file Data_Sheet_1.pdf]

Supplementary Table S1 Details of sequences used in molecular phylogenetic analysis

***Trichoderma* species**	**Strain/Voucher**	**GenBank accession number**		
		**ITS**	***rpb2***	***tef-1***
T. afarasin^{T}	CBS 130755	AY027784	–	AF348093
T. afroharzianum	NAIMCC-F-01938	KY419889	KY419895	KY419891
T. afroharzianum^{T}	GJS 04-186	FJ442265	FJ442691	FJ463301
T. aggregatum^{T}	DAOM 222156	AF443911	FJ442752	–
T. aggressivum^{T}	CBS 100526	FJ442607	KP009166	KP008993
T. alni	CPK2494	EU518652	EU498350	EU498313
T. alni ^{T}	CBS120633	EU518651	EU498349	EU498312
T. alpinum^{T}	HMAS 248821	KY687906	KY687958	KY688012
T. amazonicum	IB48	HM142356	HM142368	HM142377
T. atrobrunneum^{T}	CBS 548.92	AF443924	–	AF443942
T. atrogelatinosum^{T}	CBS 237.63	MH858272	KJ842201	–
T. bannaense^{T}	HMAS:248840	KY687923	KY687979	KY688037
T. breve^{T}	HMAS:248844	KY687927	KY687983	KY688045
T. breve	HMAS:248845	KY687928	KY687984	KY688046
T. camerunense^{T}	CBS 137272	AY027780	–	AF348107
T. catoptron^{T}	GJS 02-76	–	AY391900	AY391963
T. ceramicum^{T}	CBS 114576	FJ860743	FJ860531	FJ860628
T. christiani^{T}	CBS 132572	–	KJ665244	KJ665439
T. cinnamomeum ^{T}	GJS 97-237	–	AY391920	AY391979
T. concentricum^{T}	HMAS:248833	KY687915	KY687971	KY688027
T. concentricum	HMAS:248858	KY687941	KY687997	KY688028
T. dacrymycellum	WU 29044	FJ860749	FJ860533	FJ860633
T. epimyces^{T}	CBS 120534	EU518663	EU498360	EU498320
T. guizhouense ^{T}	CBS 131803	JN191311	JQ901400	JN215484
T. hainanense^{T}	HMAS:248837	KY687920	KY687976	KY688033
T. harzianum^{T}	CBS 226.95	AJ222720	AF545549	AY605833
T. harzianum	Thaum12	MT102390	MT118248	MT081433
T. helicolixii^{T}	CBS:133499	–	KJ665278	KJ665517
T. hengshanicum^{T}	HMAS:248852	KY687935	KY687991	KY688054
T. hirsutum^{T}	HMAS:248834	KY687916	KY687972	KY688029
T. hirsutum	HMAS:248859	KY687942	KY687998	KY688030
T. ingratum^{T}	HMAS:248822	KY687917	KY687973	KY688018
T. ingratum	HMAS:248824	KY687905	KY687964	KY688019
T. inhamatum^{T}	CBS 273.78	MH861135	FJ442725	AF348099
T. lentiforme^{T}	CBS 100542	AF469189	–	AF469195
T. liberatum^{T}	HMAS:248831	KY687913	KY687969	KY688025
T. linzhiense^{T}	HMAS:248846	KY687929	KY687985	KY688047
T. linzhiense	HMAS:248874	KY687957	KY688011	KY688048
T. lixii^{T}	CBS 110080	NR131264	KJ665290	FJ716622
T. lixii	GJS 06-94	FJ442277	FJ442800	–
T. neotropicale^{T}	CBS 130633	MH865818	–	HQ022771
T. parestonicum^{T}	CBS 120636	FJ860803	FJ860565	FJ860667
***T. phayaoense*^{T}**	**SDBR-CMU349**	**MT995122**	**MW002073**	**MW002074**
T. pleuroticola^{T}	CBS 124383	NR134420	HM142371	HM142381

T. pleuroti^{T}	CBS 124387	NR134421	HM142372	HM142382
T. pseudodensum^{T}	HMAS:248828	KY687910	KY687967	KY688023
T. pseudodensum	HMAS:248829	KY687911	KY687968	KY688024
T. pseudogelatinosum^{T}	TUFC 60186	JQ797389	JQ797405	JQ797397
T. rifaii^{T}	DIS 355B	FJ442621	FJ442720	FJ463324
T. simmonsii ^{T}	CBS 130431	AF443917	FJ442757	AF443935
T. simmonsii	GJS 92-100	AF443919	FJ442710	AF443937
T. simplex^{T}	HMAS:248842	KY687925	KY687981	KY688041
T. simplex	HMAS:248860	KY687943	KY687999	KY688042
T. solum^{T}	HMAS:248847	KY687930	KY687986	KY688045
T. solum	HMAS:248848	KY687931	KY687987	KY688050
T. tawa^{T}	GJS 97-174	AY737756	AY391956	AY392004
T. tomentosum^{T}	DAOM 178713A	DQ085432	AF545557	AF534630
T. zayuense^{T}	HMAS:248835	KY687918	KY687974	KY688031

Superscription “T” means the type species. The fungal strain obtained in this study is in bold.
