# Supplementary material for: Evaluation of a Newly Identified Endophytic Fungus, Trichoderma phayaoense for Plant Growth Promotion and Biological Control of Gummy Stem Blight and Wilt of Muskmelon
Source: Front Microbiol. 2021 Mar 5;12:634772. doi: 10.3389/fmicb.2021.634772 (PMC7973005; doi:10.3389/fmicb.2021.634772)
Supplement: Supplementary Table S2 — Treatment details in this study. [file Data_Sheet_2.pdf]

Supplementary Table S2 Treatment details in this study

Treatment number	Treatment details*
T0	Addition of 15 mL sterilized water (control)
T1	Addition of 15 mL conidial suspension of a selected endophytic fungus and 10 mL conidial suspension of *F. equiseti*
T2	Addition of 15 mL conidial suspension of a selected endophytic fungus and 10 mL conidial suspension of *S. cucurbitacearum*
T3	Addition of 15 mL conidial suspension of a selected endophytic fungus
T4	Addition of 10 mL conidial suspension of *F. equiseti*
T5	Addition of 10 mL conidial suspension of *S. cucurbitacearum*

*The conidial concentration of each fungus was 1×10^6 conidia/mL
